# The inhibitor of κB kinase β (IKKβ) phosphorylates IκBα twice in a single binding event through a sequential mechanism

**DOI:** 10.1016/j.jbc.2022.102796

**Published:** 2022-12-14

**Authors:** Anthony A. Stephenson, David J. Taggart, Guozhou Xu, Jason D. Fowler, Hao Wu, Zucai Suo

**Affiliations:** 1 The Department of Biochemistry, The Ohio State University, Columbus, Ohio, USA; 2 The Ohio State Biochemistry Program, The Ohio State University, Columbus, Ohio, USA; 3 The Department of Biochemistry, Weill Medical College of Cornell University, New York, New York, USA; 4 The Department of Biomedical Sciences, Florida State University College of Medicine, Tallahassee, Florida, USA

**Keywords:** NF-kappa B, inhibitor of NF-κB α, inhibitor of κB kinase β, pre-steady-state kinetics, protein kinases, protein phosphorylation, enzyme mechanisms, GST, glutathione-S-transferase, IκB, Inhibitor of NF-kB, IKK, Inhibitor of kB kinase, IRF, interferon regulatory factor, KD, kinase domain, NEMO, NF-κB essential modulator, NF-kB, Nuclear factor kB, TEV, tobacco etch virus, ULD, ubiquitin-like domain

## Abstract

Phosphorylation of Inhibitor of κB (IκB) proteins by IκB Kinase β (IKKβ) leads to IκB degradation and subsequent activation of nuclear factor κB transcription factors. Of particular interest is the IKKβ-catalyzed phosphorylation of IκBα residues Ser^32^ and Ser^36^ within a conserved destruction box motif. To investigate the catalytic mechanism of IKKβ, we performed pre–steady-state kinetic analysis of the phosphorylation of IκBα protein substrates catalyzed by constitutively active, human IKKβ. Phosphorylation of full-length IκBα catalyzed by IKKβ was characterized by a fast exponential phase followed by a slower linear phase. The maximum observed rate (*k*_p_) of IKKβ-catalyzed phosphorylation of IκBα was 0.32 s^−1^ and the binding affinity of ATP for the IKKβ•IκBα complex (*K*_d_) was 12 μM. Substitution of either Ser^32^ or Ser^36^ with Ala, Asp, or Cys reduced the amplitude of the exponential phase by approximately 2-fold. Thus, the exponential phase was attributed to phosphorylation of IκBα at Ser^32^ and Ser^36^, whereas the slower linear phase was attributed to phosphorylation of other residues. Interestingly, the exponential rate of phosphorylation of the IκBα(S32D) phosphomimetic amino acid substitution mutant was nearly twice that of WT IκBα and 4-fold faster than any of the other IκBα amino acid substitution mutants, suggesting that phosphorylation of Ser^32^ increases the phosphorylation rate of Ser^36^. These conclusions were supported by parallel experiments using GST-IκBα(1–54) fusion protein substrates bearing the first 54 residues of IκBα. Our data suggest a model wherein, IKKβ phosphorylates IκBα at Ser^32^ followed by Ser^36^ within a single binding event.

The nuclear factor κB (NF-κB)^2^ family of transcription factors are evolutionarily conserved master regulators of cell proliferation, innate immunity, inflammation, cell differentiation, and apoptosis (1, 2). Dysregulation of NF-κB is associated with many disorders, including cancer, autoimmune disease, neurodegenerative diseases, arthritis, and diabetes (3, 4, 5). Thus, investigation of the molecular mechanisms of NF-κB activation is important for our understanding of human disease.

The mammalian NF-κB family consists of RelA (p65), RelB, c-Rel, p50/p105 (NF-κB1), and p52/p100 (NF-κB2), which form 15 separate homodimeric or heterodimeric complexes (6). In resting cells, NF-κB dimers containing RelA, RelB, and/or c-Rel are sequestered in the cytoplasm through interactions with Inhibitor of κB (IκB) proteins IκBα, IκBβ, or IκBε. In contrast, NF-κB dimers containing p100 and p105 are localized to the cytoplasm through a C-terminal inhibitory domain containing multiple ankyrin repeat motifs. In response to various stimuli, NF-kB is activated through one of two separate pathways: the canonical or the noncanonical pathway. Within the canonical pathway, a Ser/Thr-specific IκB kinase (IKK) complex phosphorylates IκB proteins within a conserved DSGXXS destruction box motif, leading to IκB polyubiquitination, 26S proteasome-mediated degradation, and subsequent NF-κB release. Free NF-κB dimers then translocate to the nucleus to regulate transcription. In the noncanonical pathway, the IKK complex phosphorylates p100 to induce proteolytic processing of p100 to the activated NF-κB2 subunit p52, which also localizes to the nucleus. The IKK complex also catalyzes the phosphorylation of several protein substrates within alternative signaling pathways, and this activity is thought to coordinate the functions of the NF-κB pathways with other cellular pathways (reviewed in reference (7)).

The IKK complex consists of the nonenzymatic protein NEMO (NF-κB essential modulator, also called IKKγ) and a homodimer or heterodimer of the catalytic subunits IKKα and IKKβ (8, 9, 10, 11, 12). Although IKKα and IKKβ share 54% amino acid sequence identity, these kinases possess distinct substrate specificities. For example, IKKα predominantly catalyzes phosphorylation of p100 within the noncanonical pathway (13, 14). In contrast, IKKβ is primarily responsible for the phosphorylation of IkBα, IκBβ, IκBε within the canonical pathway (15, 16, 17).

Structural studies indicate that IKKβ possesses a trimodular architecture (Fig. 1*A*) consisting of an N-terminal kinase domain (KD), a ubiquitin-like domain (ULD), and a C-terminal scaffold/dimerization domain (18, 19, 20). The KD contains an activation loop with the MEK consensus motif SxxxS (S177 and S181 in human IKKβ, Fig. 1*A*). This activation loop is essential for the activity of IKKβ as mutational analysis indicates that changing these Ser residues to Ala prevents IKKβ activation, whereas substitution of these critical Ser residues with phosphomimetic Glu residues renders the kinase constitutively active (12, 19, 21, 22).

**Figure 1:**
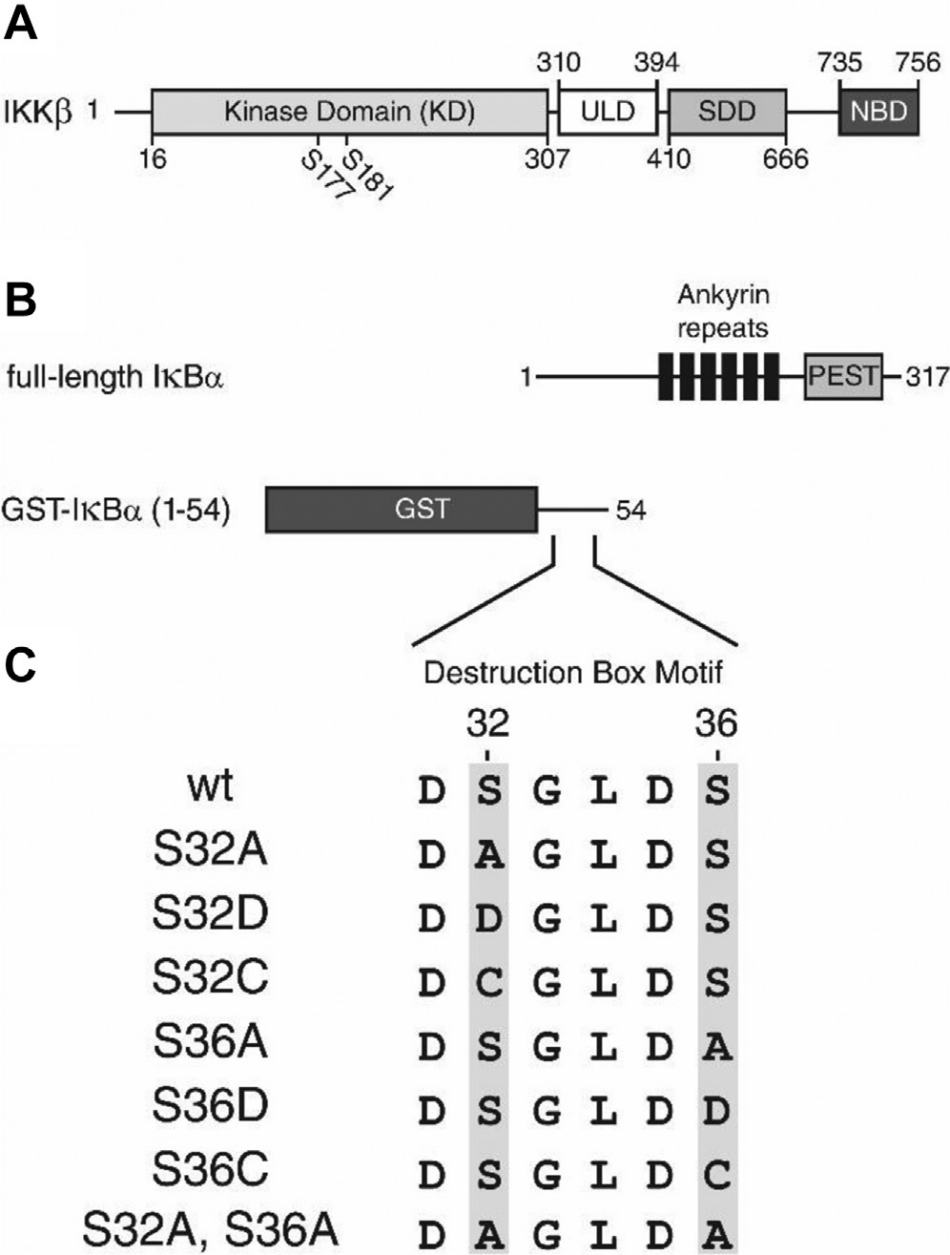
**Schematic diagrams of human IKKβ, IκBα, and IκBα mutations.***A*, human IKKβ. The boundaries of the kinase domain (KD), Ubiquitin-like domain (ULD), Scaffold/Dimerization domain (SDD), and NEMO-binding domain (NBD) are shown. The positions of Ser^177^ and Ser^181^ within the kinase activation loop are also indicated. *B*, full-length IκBα and the GST-IκBα(1–54) fusion proteins. IκBα is composed of the signal response domain (SRD) (residue 1–66), the ankyrin repeat domain (ARD) (residue 67–280), and the PEST domain (residue 281–317). *C*, IκBα amino acid substitution mutants. The destruction box motif (residues 31–36) of wt IκBα and each IκBα amino acid substitution mutation are shown. The specific sites of IκBα phosphorylation are highlighted in *gray*. IκB, inhibitor of IκB; IKKβ, IκB Kinase β.

Although the phosphorylation of IκB proteins by IKKβ is a critical step within the canonical pathway of NF-κB activation, the molecular mechanism of IKKβ is poorly understood. Here, we used pre–steady-state kinetic analysis to investigate the molecular mechanism of IκBα phosphorylation catalyzed by constitutively active IKKβ. Our data indicate that IKKβ phosphorylates full-length IκBα twice within the conserved DSGXXS destruction box motif (Fig. 1*C*) during a single binding event. Our study also suggests that IKKβ preferentially phosphorylates IκBα sequentially at Ser^32^, followed by Ser^36^. We conclude that these aspects of the kinetic mechanism of IKKβ may be shared among the IKK and IKK-related kinases.

## Results

### Determination of the pre–steady-state kinetic parameters of IKKβ phosphorylation of full-length IκBα

The activity of human IKKβ (Fig. 1*A*) is significantly increased upon phosphorylation of activation loop residues Ser^177^ and Ser^181^ within the kinase domain (21, 22). In order to measure the catalytic rates of activated IKKβ, we chose to utilize a constitutively active IKKβ (S177E, S181E) phosphomimetic amino acid substitution mutation (12, 19, 21) for our study. Within the canonical pathway of NF-κB activation, IKKβ specifically phosphorylates IκBα at residues Ser^32^ and Ser^36^ (Fig. 1, *B* and *C*) to induce the proteolytic degradation of IκBα and the subsequent release of NF-κB dimers. Importantly, IκBα is also phosphorylated at other sites *in vivo*, particularly within the C-terminal PEST domain (Fig. 1*B*) (23, 24). However, unlike phosphorylation of residues within the destruction box motif, phosphorylation of residues within the PEST domain does not specifically target IκBα for ubiquitination and 26S proteasome-mediated degradation (24).

**Figure 2:**
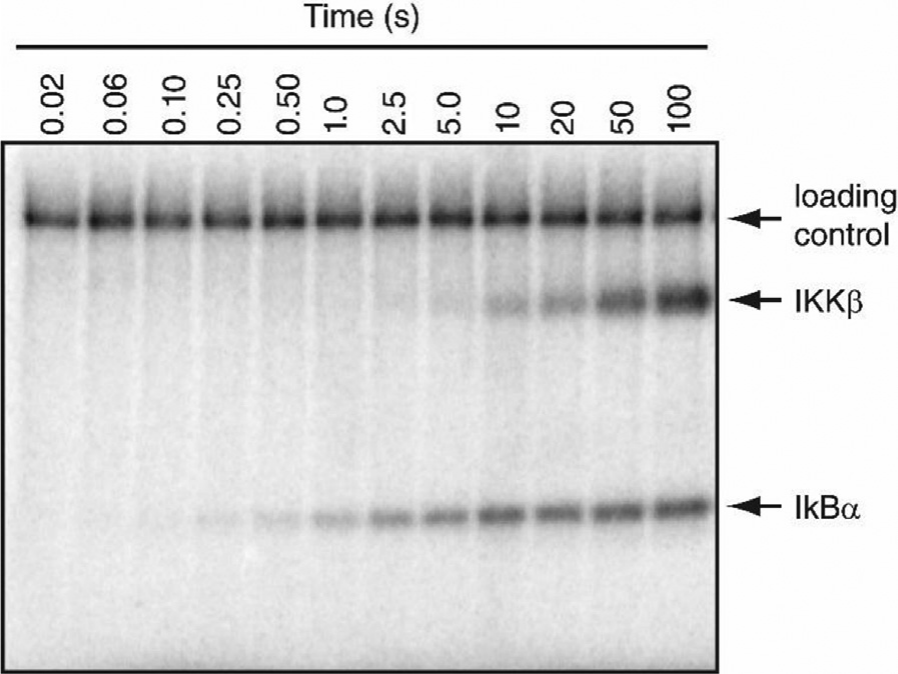
**Example autoradiogram demonstrating IκBα phosphorylation catalyzed by IKKβ.** A preincubated solution of IKKβ (3 μM) and full-length IκBα (0.75 μM) was rapidly mixed with a solution containing a [γ-^32^P]-labeled loading control and 500 μM [γ-^32^P]ATP. After various times, the reactions were stopped by the addition of EDTA to a final concentration of 375 mM. The reaction products were then resolved by using SDS-PAGE and visualized by autoradiography. The positions of the radiolabeled loading control, IKKβ, and IκBα are indicated. IκB, inhibitor of IκB; IKKβ, IκB Kinase β.

Steady-state kinetic analysis has demonstrated that IKKβ phosphorylates IκBα by using a random sequential mechanism (25), indicating that IKKβ can bind IκBα and ATP in any order prior to catalysis. However, because IKKβ exhibits autophosphorylation (Fig. 2), we chose to measure the pre–steady-state kinetic parameters of IKKβ-catalyzed phosphorylation of IκBα by first incubating IKKβ with the IκBα protein substrate to generate the IKKβ•IκBα complex and subsequently initiating the reaction by the rapid addition of ATP. To initially characterize the molecular mechanism of IKKβ-catalyzed phosphorylation of full-length IκBα, the ground-state–binding affinity of ATP (*K*_d_) for the IKKβ•IκBα complex and the maximum observed rate (*k*_p_) of IKKβ-catalyzed phosphorylation were determined by using pre–steady-state kinetic analysis. To this end, we measured the ATP concentration dependence of the phosphorylation rate of IKKβ under conditions where IKKβ was in a 4-fold molar excess compared to the IκBα substrate to ensure that nearly all of the IκBα substrate was initially bound by the kinase. IKKβ has previously been shown to bind IκBα with an equilibrium dissociation constant (*K*_d_) of 56 nM (26), and thus, we calculated that >98% of the IκBα substrate was initially bound by IKKβ under our experimental conditions.

**Figure 3:**
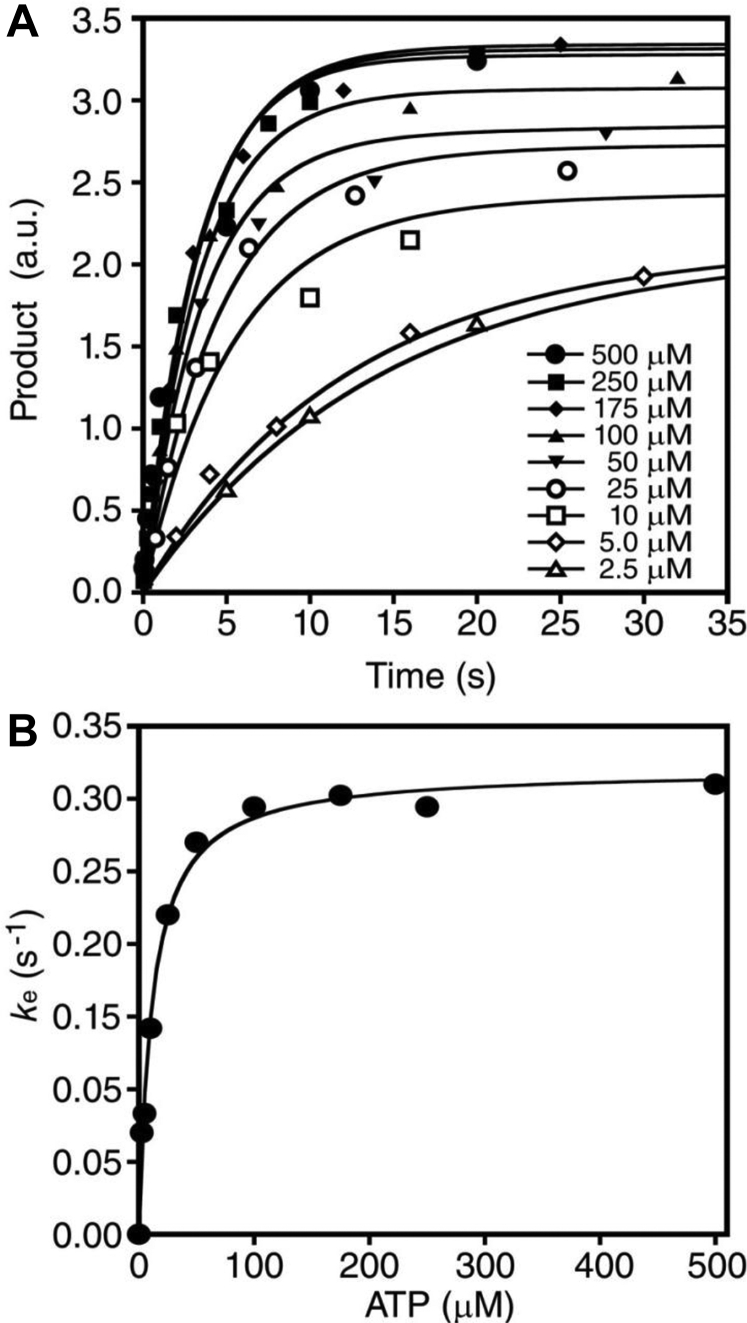
**ATP concentration dependence of the pre–steady-state rate of full-length IκBα phosphorylation catalyzed by constitutively active IKKβ.***A*, a preincubated solution of IKKβ (3 μM) and full-length IκBα (0.75 μM) was rapidly mixed with a solution containing a [γ-^32^P]-labeled loading control and increasing concentrations of [γ-^32^P]ATP for various times. The *solid lines* represent the best fits to biphasic Equation 1 (see Experimental procedures). *B*, the exponential rates (*k*_e_) obtained from the data fitting above were plotted as a function of ATP concentration and fit to hyperbolic Equation 2, yielding a *k*_p_ of 0.32 ± 0.01 s^-1^ and a *K*_d_ of 12 ± 1 μM. IκB, inhibitor of IκB; IKKβ, IκB Kinase β.

**Figure 4:**
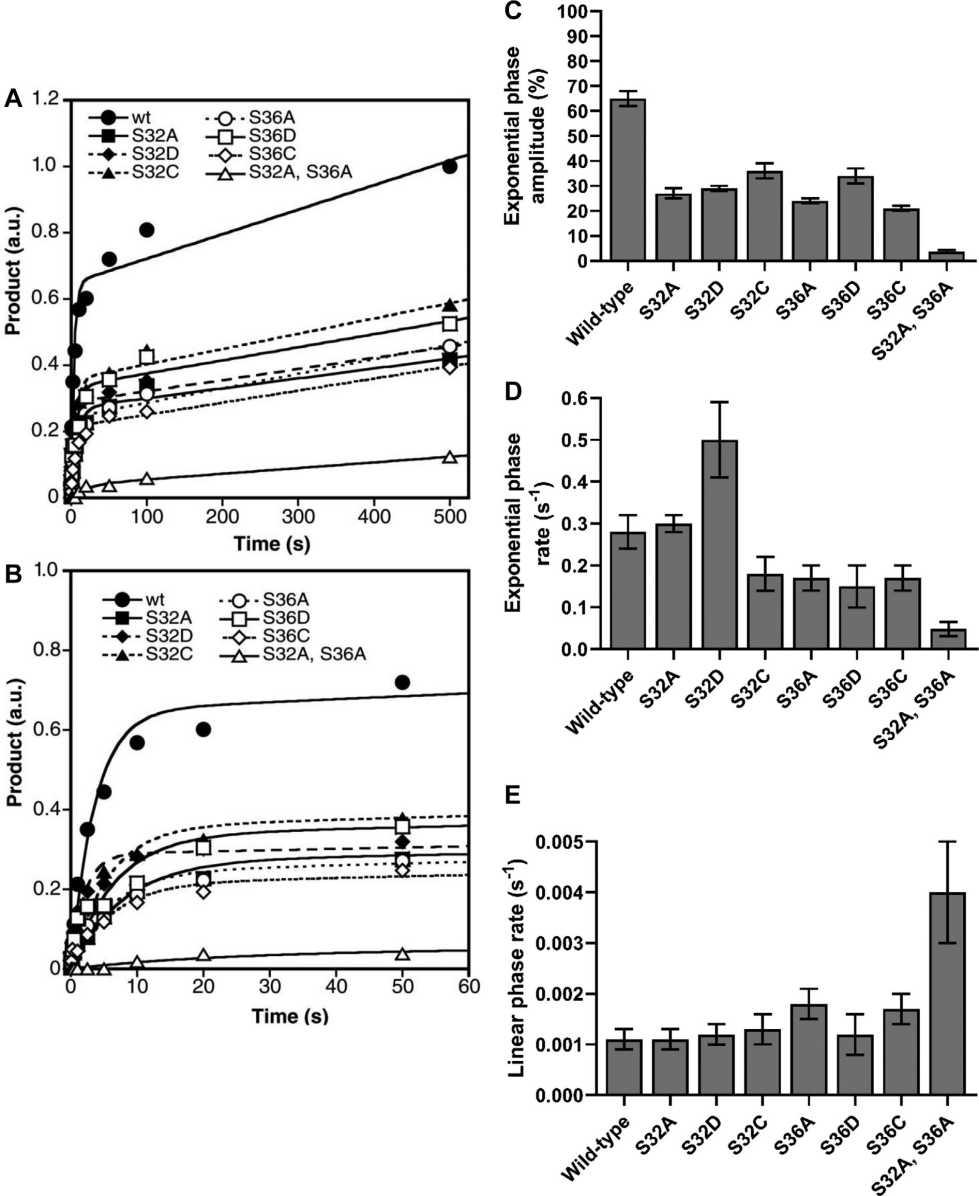
**IKKβ phosphorylation of full-length IκBα and full-length IκBα amino acid substitution mutants.** A preincubated solution of IKKβ (3 μM) and the indicated full-length IκBα (0.75 μM) was rapidly mixed with a solution containing a [γ-^32^P]-labeled loading control and [γ-^32^P]ATP (500 μM) for various times. Phosphorylation of the full-length IκBα substrates was then normalized to the loading control and plotted as arbitrary phosphorylation units (a.u.) as a function of time from (*A*) 0 to 500 s or (*B*) 0 to 50 s. The *solid lines* represent the best fits to biphasic Equation 1 (see Experimental procedures). The exponential phase amplitudes (*C*), the exponential phase rates (*D*), and the linear phase rates (*E*) were separately plotted against WT IκBα and its various mutants. IκB, inhibitor of IκB; IKKβ, IκB Kinase β.

A preincubated solution of full-length IκBα and IKKβ (S177E, S181E) was rapidly mixed with a solution containing increasing concentrations of [γ-^32^P]ATP for various times. The reaction products were then separated by SDS-PAGE and phosphorylation of IκBα was quantified by using autoradiography (Fig. 2). A [γ-^32^P]-labeled, linearized DNA vector was included as a loading control because it was easily separated from both IKKβ and IκBα by SDS-PAGE, and it provided a consistent signal after incubation with IKKβ, IκBα, and ATP (Fig. 2). The amount of phosphorylated IκBα detected at each time point was normalized to the loading control and plotted as a function of reaction time (Fig. 3*A*). The resulting plots indicated that IKKβ (S177E, S181E) phosphorylated IκBα with an initial, fast exponential phase rate (*k*_e_), followed by a significantly slower linear phase rate (*k*_l_) and therefore, these data were fit to biphasic Equation 1 (see Experimental procedures). The biphasic nature of the plot can be clearly observed in Figure 4*A* when longer reaction time points were included in the plot. We hypothesized that the initial exponential rate corresponded to phosphorylation of IκBα at residues Ser^32^ and Ser^36^ within the destruction box motif, and the significantly slower linear rate resulted from either the phosphorylation of IκBα at sites outside of the destruction box motif, such as the PEST domain (Fig. 1*B*), or phosphorylation of IκBα substrate that was not initially bound in a productive complex by IKKβ, or both. The exponential rates of IKKβ-catalyzed phosphorylation of IκBα were then plotted as a function of ATP concentration and fit to hyperbolic Equation 2 (Fig. 3*B*), yielding a *k*_p_ of 0.32 ± 0.01 s^−1^ for the maximum observed rate of IKKβ-catalyzed phosphorylation of full-length IκBα and a *K*_d_ of 12 ± 1 μM for ATP binding to the IKKβ•IκBα complex.

### IKKβ phosphorylates IκBα twice within a single binding event

To further investigate the molecular mechanism of IKKβ phosphorylation, we sought to test the ability of IKKβ (S177E, S181E) to phosphorylate either Ser^32^ or Ser^36^ within the destruction box motif of IκBα (Fig. 1, *B* and *C*). To this end, we evaluated the phosphorylation of seven separate full-length IκBα amino acid substitution mutants (Fig. 1*C*) in which either residue Ser^32^ or Ser^36^ were individually changed to (i) Ala to eliminate the phosphorylation site, (ii) Asp to mimic the size and charge of a phosphorylated Ser, or iii) Cys to mimic the size and hydrophilic character of Ser while eliminating the phosphorylation site. An IκBα(S32A, S36A) double alanine substitution mutant was also tested in which both Ser residues within the destruction box motif were eliminated. The amount of phosphorylated IκBα product was plotted as a function of reaction time and fit to biphasic Equation 1 (Fig. 4). Kinetic data derived from the best fit curves are reported in Figure 4, *C*–*E* and Table 1.

**Table 1:** Kinetic parameters^a^ of full-length IκBα phosphorylation by IKKβ

Full-length IκBα substrate	Cognate phosphorylation sites	Exponential phase amplitude (%)	Exponential rate (s^-1^)	Linear rate (s^-1^)
WT	S32, S36	65 ± 3	0.28 ± 0.04	0.0011 ± 0.0002
S32A	S36	27 ± 2	0.13 ± 0.02	0.0011 ± 0.0002
S32D	S36	29 ± 1	0.50 ± 0.09	0.0012 ± 0.0002
S32C	S36	36 ± 3	0.18 ± 0.04	0.0013 ± 0.0003
S36A	S32	24 ± 1	0.17 ± 0.03	0.0018 ± 0.0003
S36D	S32	34 ± 3	0.15 ± 0.05	0.0012 ± 0.0004
S36C	S32	21 ± 1	0.17 ± 0.03	0.0017 ± 0.0003
S32A, S36A	none	3.8 ± 0.6	0.048 ± 0.017	0.004 ± 0.001

a Phosphorylation of the full-length IκBα substrates by IKKβ (S177E, S181E) were fit to Equation 1, [Product] = A[1 – exp(-*k*_e_t)] + *k*_l_t where A is the exponential phase amplitude, *k*_e_ is the exponential rate and *k*_l_ is the linear rate (see Experimental procedures).

Comparing the kinetic parameters of IKKβ-catalyzed phosphorylation of wt IκBα and the IκBα amino acid substitution mutants, the amplitude of the exponential phase produced by phosphorylation of each of the IκBα single amino acid substitution mutants was reduced approximately 2-fold when compared to the exponential phase amplitude of wt IκBα (Fig. 4*C* and Table 1). Additionally, the observed exponential phase amplitude produced by phosphorylation of the IκBα(S32A, S36A) double amino acid substitution mutant was reduced by 17-fold when compared to wt IκBα (Fig. 4*C* and Table 1). These data indicate that the exponential phase of IKKβ-catalyzed phosphorylation of IκBα corresponds to phosphorylation of IκBα at Ser^32^ and Ser^36^. Interestingly, the exponential phase of wt IκBα phosphorylation can be fit to a single rate (Fig. 4), indicating that IKKβ phosphorylated IκBα at Ser^32^ and Ser^36^ rapidly, without a significant intervening step. This finding strongly suggests that IKKβ phosphorylated wt IκBα twice, without complete disassociation and reassociation of the IKKβ•IκBα complex. Thus, we concluded that IKKβ phosphorylates IκBα processively within a single binding event.

Interestingly, the exponential rate of phosphorylation of the IκBα(S32D) phosphomimetic substitution mutant was increased by approximately 1.8 fold when compared to the exponential rate of phosphorylation of wt IκBα (Fig. 4*D* and Table 1). In contrast, the exponential rate of phosphorylation for all of the other IκBα single amino acid substitution mutants, including the IκBα(S36D) phosphomimetic amino acid substitution mutant, was reduced by approximately 1.5-fold when compared to wt IκBα phosphorylation (Fig. 4*D* and Table 1). These data suggest that if IκBα is phosphorylated at Ser^32^, the rate at which IKKβ catalyzes the second phosphorylation event at Ser^36^ is increased, while phosphorylation of IκBα Ser^36^ does not increase the rate of phosphorylation at Ser^32^.

The linear rate of IKKβ-catalyzed phosphorylation of the IκBα single amino acid substitution mutants was virtually identical (within 2-fold) to the linear rate of IKKβ-catalyzed phosphorylation of wt IκBα (Fig. 4*E* and Table 1). These results are consistent with the hypothesis that the linear phase of phosphorylation of IκBα and the IκBα amino acid substitution mutants results primarily from phosphorylation of IκBα at sites outside of the destruction box motif (Fig. 1, *B* and *C*) by IKKβ. Interestingly, the linear rate of IKKβ-catalyzed phosphorylation of the IκBα(S32A, S36A) double amino acid substitution mutant was 2- to 4-fold faster than for wt IκBα and the IκBα single amino acid substitution mutants (Fig. 4*E* and Table 1), suggesting that IKKβ-catalyzed phosphorylation of Ser^32^ and Ser^36^ may have slowed the linear PEST domain phosphorylation rate in these experiments.

### Interactions between IKKβ and the C-terminal region of IκBα do not significantly influence the molecular mechanism of IKKβ-catalyzed phosphorylation

**Figure 5:**
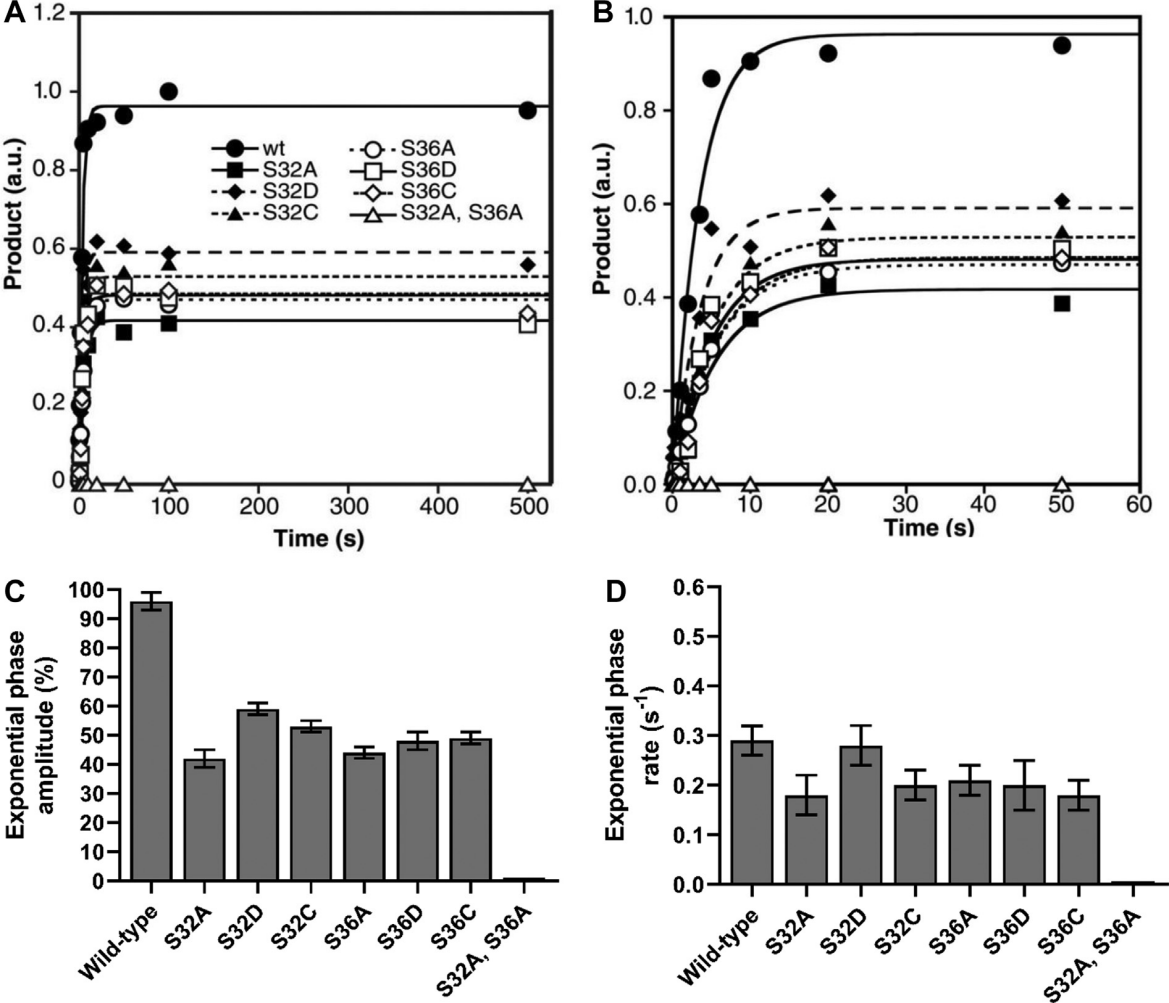
**IKKβ phosphorylation of GST-IκBα(1–54) and GST-IκBα(1–54) amino acid substitution mutants.** A preincubated solution of IKKβ (3 μM) and the indicated GST-IκBα(1–54) fusion protein (0.75 μM) was rapidly mixed with a solution containing a [γ-^32^P]-labeled loading control and [γ-^32^P]ATP (500 μM) for various times. Phosphorylation of the full-length IκBα substrates was then normalized to the loading control and plotted as arbitrary phosphorylation units (au) as a function of time from (*A*) 0 to 500 s or (*B*) 0 to 50 s. The *solid lines* represent the best fits to single-exponential Equation 3 (see Experimental procedures). The exponential phase amplitudes (*C*) and the exponential phase rates (*D*) were separately plotted against WT IκBα and its various mutants. IκB, inhibitor of IκB; IKKβ, IκB Kinase β.

Although IKKβ preferentially phosphorylates IκBα within the destruction box motif (Fig. 1, *B* and *C*), we predicted that IKKβ is also capable of phosphorylating IκBα at sites within the C-terminal region (residues 55–317), such as within the PEST domain (Fig. 1*B*). To rule out the possibility that the observed rates of IKKβ-catalyzed phosphorylation of full-length IκBα were significantly influenced by phosphorylation of IκBα residues outside of the destruction box motif, we evaluated the phosphorylation of glutathione-S-transferase (GST)-IκBα(1–54) fusion proteins (Fig. 1, *B* and *C*). These GST-IκBα(1–54) protein substrates lack the C-terminal region of IκBα. Thus, we predicted that nonspecific substrate phosphorylation by IKKβ would be reduced when testing these protein substrates. We assessed the IKKβ-catalyzed phosphorylation of GST-IκBα(1–54) bearing the first 54 residues of IκBα, including the destruction box motif, and seven separate amino acid substitution mutations of GST-IκBα(1–54) (Fig. 1, *B* and *C*) under pre–steady-state conditions. The amount of phosphorylated IκBα substrate detected was plotted as a function of reaction time (Fig. 5, *A* and *B*). In contrast to phosphorylation of the full-length IκBα substrates (Fig. 4, *A* and *B*), only a single exponential phase of GST-IκBα(1–54) was observed (Fig. 5, *A* and *B*). Therefore, these data were fit to single-exponential Equation 3 (see Experimental procedures). Kinetic data derived from the best fit curves are reported in Figure 5, *C* and *D* as well as Table 2. The absence of a clear linear phase was likely due to either a reduction in nonspecific phosphorylation of the GST-IκBα(1–54) fusion protein at sites outside of the destruction box motif when compared to phosphorylation of the full-length IκBα substrates and/or a reduction in the number of initial, unproductive protein complexes. Consistently, the rate of GST-IκBα(1–54)(S32A, S36A) phosphorylation was too slow to be accurately measured (Fig. 5, *A* and *B*).

**Table 2:** Kinetic parameters of GST-IκBα(1–54)^a^ phosphorylation by IKKβ

GST-IκBα(1–54) substrate	Cognate phosphorylation sites	Exponential phase amplitude (%)	Exponential rate (s^-1^)
WT	S32, S36	96 ± 3	0.29 ± 0.03
S32A	S36	42 ± 3	0.18 ± 0.04
S32D	S36	59 ± 2	0.28 ± 0.04
S32C	S36	53 ± 2	0.20 ± 0.03
S36A	S32	44 ± 2	0.21 ± 0.03
S36D	S32	48 ± 3	0.20 ± 0.05
S36C	S32	49 ± 2	0.18 ± 0.03
S32A, S36A^b^	none	–	–

a Phosphorylation of the GST- IκBα(1–54) substrates by IKKβ (S177E, S181E) were fit to Equation 3, [Product] = A[1 – exp(-*k*_e_t)] where A is the exponential phase amplitude and *k*_e_ is the exponential rate (see Experimental procedures).

b A dash (-) indicates that phosphorylation of GST-IκBα(1–54)(S32A, S36A) by IKKβ was too low to be accurately quantified.

**Figure 6:**
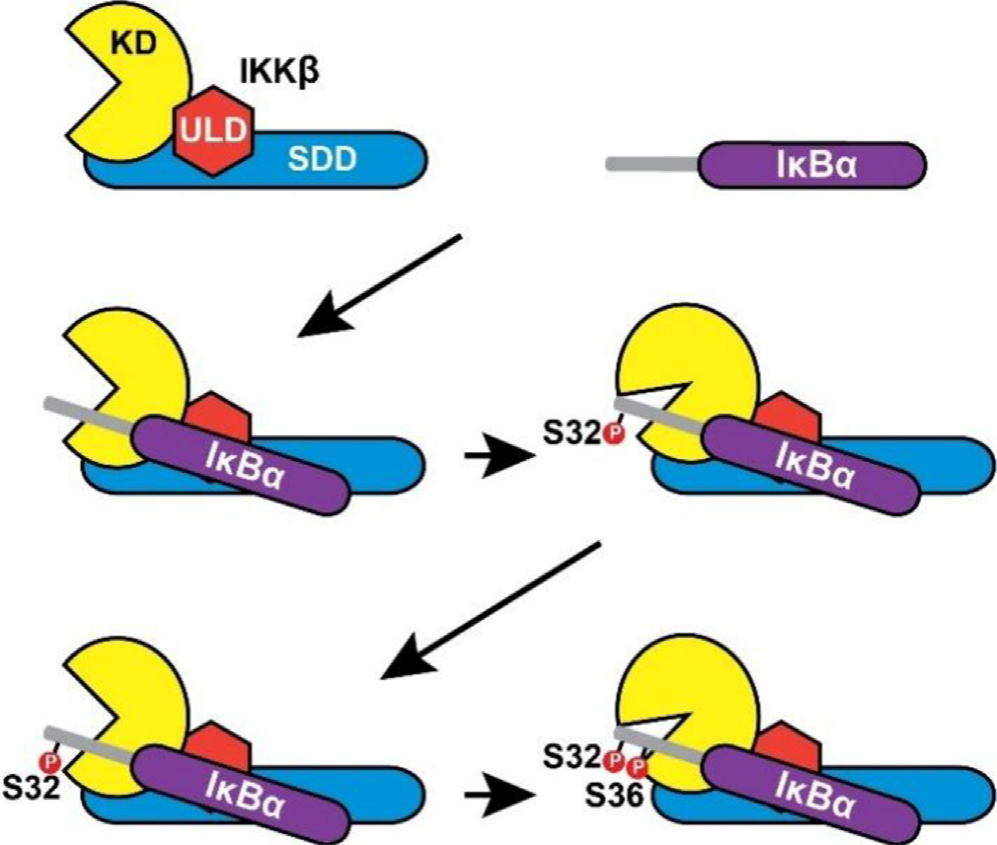
**Proposed model of double IκBα phosphorylation catalyzed by IKKβ within a single binding event.** The C-terminal region of IκBα is first bound by the ULD and SDD of IKKβ, which serves to coordinate the N-terminal region of IκBα for phosphorylation by the KD. After recognition and binding of the IκBα phosphorylation site by the KD, IKKβ catalyzes the first phosphorylation of IκBα. The N-terminal region of IκBα is then rapidly repositioned within the kinase active site and the second phosphorylation event is catalyzed, while the C-terminal region of IκBα remains bound to IKKβ. IκB, inhibitor of IκB; IKKβ, IκB Kinase β; KD, kinase domain; ULD, ubiquitin-like domain; SDD, scaffold/dimerization domain.

It has been previously demonstrated that the C-terminal region (residues 55–317) of IκBα (Fig. 1*B*) interacts with the ULD and SSD of IKKβ (Fig. 1*A*) (19). Such interaction may help to properly position the N-terminal destruction box motif of IκBα for phosphorylation by the KD of IKKβ (Fig. 6) and thus, influence the molecular mechanism of IKKβ. Interestingly, in agreement with our observations of IKKβ-catalyzed phosphorylation of the full-length IκBα substrates, the amplitude of the exponential phase of GST-IκBα(1–54) wt phosphorylation was approximately 2-fold greater than the exponential phase amplitude of all six of the GST-IκBα(1–54) single amino acid substitution mutants (Fig. 5*C* and Table 2). Thus, we concluded that interactions between IKKβ and the C-terminal region of IκBα are not required for IKKβ to phosphorylate IκBα twice within a single binding event. Furthermore, the full-length IκBα and the GST-IκBα(1–54) wt substrates were both phosphorylated at similar exponential rates (Figs. 4*D* and 5*D*, Tables 1 and 2), suggesting that interactions between IKKβ and the C-terminal domain of IκBα do not significantly alter the overall exponential rate of IKKβ-catalyzed phosphorylation. However, in contrast to the results obtained with the full-length IκBα amino acid substitution mutants (Fig. 4*D* and Table 1), the GST-IκBα(1–54)(S32D) phosphomimetic amino acid substitution mutant was phosphorylated at a similar rate to the GST-IκBα(1–54) wt substrate, and the remaining GST-IκBα(1–54) single amino acid substitution mutants were phosphorylated at a rate that was only reduced by approximately 1.5-fold when compared to GST-IκBα(1–54) wt (Fig. 5*D* and Table 2). Thus, substitution of destruction box residues Ser^32^ or Ser^36^ of the GST-IκBα(1–54) substrates altered the exponential rate of phosphorylation of these truncated substrates to a lesser extent than substitution of Ser^32^ or Ser^36^ within the full-length IκBα substrates. We concluded that although interactions between IKKβ and the C-terminus of IκBα do not significantly influence the overall exponential rate of IKKβ-catalyzed phosphorylation, these interactions may influence the individual rates of phosphorylation of IκBα Ser^32^ and Ser^36^.

## Discussion

The pathways of NF-κB regulation are widely viewed as potential therapeutic targets (27, 28). Thus, an understanding of the molecular mechanisms of the enzymes involved in the regulation of these critical pathways, such as IKKβ, may be advantageous for the development of novel therapies for the treatment of a wide array of human diseases, such as cancer. The IKKβ-catalyzed phosphorylation of IκB protein substrates is an essential step within the canonical pathway of NF-κB activation. Thus, the IKKβ signal transduction pathway has been the subject of intensive study (29). However, despite recent crystal structures of IKKβ (18, 19, 20), the molecular mechanism of IKKβ-catalyzed phosphorylation remains poorly understood. Here we employed a pre–steady-state kinetic approach to investigate the phosphorylation of IκBα by a constitutively active form of human IKKβ. Importantly, we chose to study the phosphorylation of both full-length IκBα and GTS-IκBα(1–54) fusion protein substrates (Fig. 1, *B* and *C*) to investigate potentially important interactions between IKKβ and the C-terminal domain of IκBα.

We concluded that IKKβ phosphorylates IκBα twice within a single binding event and that phosphorylation of Ser^32^ of IκBα increases the rate of phosphorylation of Ser^36^. Thus, we propose a model (Fig. 6) wherein IKKβ binds IκBα and processively phosphorylates IκBα first at Ser^32^, followed by a second phosphorylation event at Ser^36^. Reciprocal GST pull-down experiments have indicated that the ULD and SSD of IKKβ bind strongly to the C-terminal region (residues 55–317, Fig. 1*B*) of IκBα (19). Such a binding event may orient the N-terminus of IκBα for proper phosphorylation at Ser^32^ and Ser^36^ by the kinase domain of IKKβ. Consistently, deletion of the ULD-scaffold/dimerization domain of IKKβ results in the shifting of phosphorylation specificity from the cognate sites Ser^32^ and Ser^36^ of IκBα to its C-terminal PEST domain (Fig. 1*B*) (19). Besides the model in Figure 6, IKKβ may phosphorylate Ser^36^ first, followed by Ser^32^, or randomly on either Ser^32^ or Ser^36^ first. Our kinetic data (Fig. 4 and Table 1) indicate that the model in Figure 6, relative to the two competing models, will allow IKKβ to achieve the highest IκBα phosphorylation efficiency.

**Figure 7:**
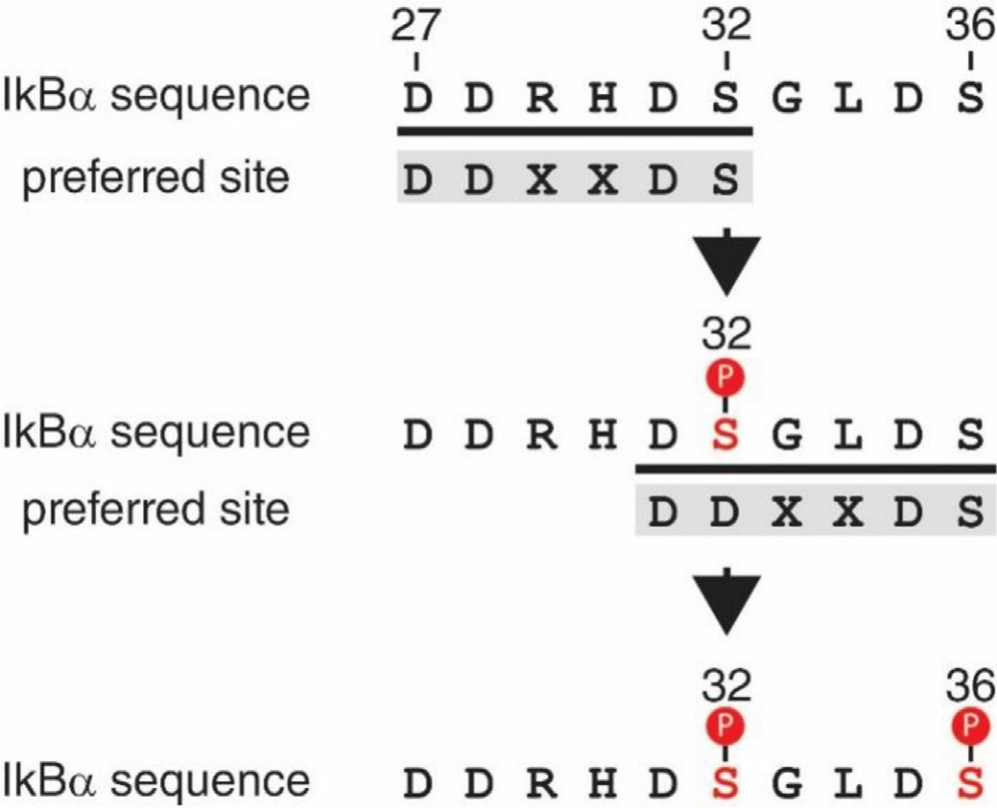
**Model of sequential IκBα phosphorylation catalyzed by IKKβ.** Residues 27 to 36 of IκBα are shown above with the proposed, preferred recognition site of IKKβ shown below. After the first phosphorylation event, the size and charge of phosphorylated Ser^32^ mimics Asp, thereby changing residues 31 to 36 into a second preferred recognition site. IκB, inhibitor of IκB; IKKβ, IκB Kinase β.

Furthermore, of the two cognate IκBα phosphorylation sites, the IKKβ kinase domain may preferentially phosphorylate IκBα at Ser^32^ first by virtue of a preference for phosphorylation of the 27-DDXXDS-32 amino acid sequence (Fig. 7). After phosphorylation of IκBα at Ser^32^, the phosphorylated Ser^32^ residue will possess a similar size and charge as an aspartic acid residue. Thus, after phosphorylation of Ser^32^, the amino acid sequence surrounding Ser^36^ will also resemble the potentially preferred amino acid sequence (Fig. 7). Binding of the IκBα C-terminal region by IKKβ may be important for this preferential phosphorylation step as the exponential rate of phosphorylation of the full-length IκBα(S32D) phosphomimetic substitution mutant was increased by approximately 2-fold with respect to the IκBα wt substrate (Fig. 4*D* and Table 1), whereas this increase in the exponential rate of phosphorylation was not observed with the GST-IκBα(1–54)(S32D) fusion protein substrate, which lacks the C-terminal region of IκBα (Fig. 5*D* and Table 2). Importantly, this sequential phosphorylation of Ser^32^ followed by Ser^36^ is not a requirement for IKKβ activity, as phosphorylation of Ser^32^ and Ser^36^ can occur independently of one another (Figs. 4 and 5). However, it is clear that IKKβ is sensitive to even minor changes in the amino acid sequence of the IκBα destruction box motif as evidenced by the fact that the exponential rate of phosphorylation of every IκBα amino acid substitution mutant, except for the phosphomimetic S32D substitution, was slower than the exponential rate of phosphorylation of IκBα substrates bearing the WT destruction box motif (Tables 1 and 2).

IKKβ must phosphorylate IκBα at both Ser^32^ and Ser^36^ to induce the ubiquitination and degradation of IκBα and the subsequent release of NF-κB dimers. Kinases are known to phosphorylate protein substrates at multiple sites through one of two separate mechanisms (reviewed in reference (30)): (i) a distributive mechanism, in which a kinase phosphorylates a substrate only once per binding event or (ii) a processive mechanism, in which a kinase phosphorylates a substrate two or more times per binding event. If IKKβ utilized a distributive mechanism to phosphorylate IκBα, then we would predict that pre–steady-state analysis would reveal separate rates for the first and second phosphorylation events due to the intervening disassociation and reassociation steps. Therefore, our finding that the exponential phase of IKKβ-catalyzed phosphorylation of full-length IκBα can be fit to a single rate (Fig. 4) is most consistent with the processive phosphorylation of Ser^32^ and Ser^36^ without a significant intervening step. In support of this conclusion, the amplitude of the exponential phase produced by phosphorylation of each of the single amino acid substitution mutants in the IκBα destruction box motif was reduced approximately 2-fold when compared to the exponential phase amplitude of wt IκBα (Fig. 4*C* and Table 1).

Single Ser to Ala amino acid substitutions at Ser^32^ or Ser^36^ allow IκBα to escape polyubiquitination and degradation and render IκBα a constitutive inhibitor of NF-κB *in vivo* (31, 32, 33, 34). Thus, a single phosphorylation event catalyzed by IKKβ is insufficient to activate NF-κB. The catalysis of two phosphorylation events within a single binding event is an efficient mechanism by which IKKβ may activate NF-κB. Strikingly, IKKβ is known to catalyze two separate phosphorylation events with multiple substrates, including IκBα, IκBβ, and IκBε (reviewed in reference (35)), suggesting that the processive catalysis of two phosphorylation events may be a general mechanism by which IKKβ phosphorylates protein substrates. Additionally, IKKα is known to phosphorylate p100/NF-κB2 at multiple residues to induce the proteolytic activation of this NF-κB subunit (13) and the IKK-related kinases, IKKε (also called IKK-i) and TANK-binding kinase 1, are known to phosphorylate the interferon regulatory factor (IRF) proteins IRF-3, IRF-5, and IRF-7 at two or more sites to activate these critical regulators of the innate immunity response (reviewed in reference (36)). Thus, the processive catalysis of multiple phosphorylation events during a single binding event may be a conserved mechanism of both the IKK and IKK-related kinases.

## Experimental procedures

### Materials

Reagents were purchased from the following companies: [γ-^32^P]ATP from MP Biomedicals, ATP from Thermo Fisher Scientific, Optikinase from USB corporation, and plasmid pBluescript II KS + from Agilent Technologies.

### Expression and purification of constitutively active human IKKβ (S177E, S181E)

A recombinant baculovirus was generated by using the Bac-to-Bac system (Invitrogen) to express full-length, human IKKβ (S177E, S181E) (residues 1–756) bearing a C-terminal hexahistidine affinity purification tag. The recombinant kinase was produced by infection of Hi5 insect cells in SF-900 II serum-free media (Gibco) in suspension. At 48 h postinfection, the cells were harvested and recombinant IKKβ (S177E, S181E) was purified as previously described (19). Briefly, Hi5 cell pellets expressing IKKβ were suspended in buffer A (20 mM Tris, pH 8.0, 200 mM NaCl, 10 mM imidazole, and 0.25 mM tris(2-carboxyethyl)phosphine), disrupted by sonication and cleared by centrifugation at 40,000*g* for 1 h. The cleared cell lysate was then incubated with Ni-NTA resin (Qiagen) and unbound proteins were removed by extensive washing with Buffer A. Bound proteins were subsequently eluted with a gradient of 10 mM to 250 mM imidazole with buffer B (20 mM Tris, pH 8.0, 200 mM NaCl, 250 mM imidazole, and 0.25 mM tris(2-carboxyethyl)phosphine). IKKβ-containing fractions were then pooled and applied to a Resource-Q anion exchange column (GE healthcare). The column was washed with buffer C (40 mM Tris, pH 8.0, 0.1 M NaCl, 2 mM EDTA, 5 mM DTT) and bound proteins were eluted using a linear gradient of 0.1 M to 1 M NaCl with buffer D (40 mM Tris, pH 8.0, 2 mM EDTA, 5 mM DTT, 1 M NaCl). Fractions containing IKKβ were pooled, concentrated, and applied to a gel filtration column equilibrated to buffer F (20 mM Tris, pH 7.6, 150 mM NaCl, 10 mM DTT). The gel filtration fractions containing IKKβ were then concentrated and finally dialyzed against storage buffer (50 mM Tris–Cl, pH 7.6, 100 mM NaCl, 1 mM DTT, 0.2 mM EDTA, 50% glycerol). Full-length IKKβ (S177E, S181E) was determined to be >95% pure on the basis of Coomassie staining of SDS-PAGE gels.

### Expression and purification of full-length IκBα protein substrates

Full-length, human IκBα was produced as a GST-fusion protein in *Escherichia coli* by using vector pGEX4T3/IκBα wt (19). The GST-IκBα fusion protein possessed an N-terminal GST tag, followed by a tobacco etch virus (TEV) protease cleavage site and residues 1 to 317 of IκBα. GST-tagged IκBα was expressed in BL21 (DE3) pLysS cells at 20 °C for 16 h. The cells were then harvested, suspended in GST-binding buffer (25 mM NaH_2_PO_4_, pH 7.3, 140 mM NaCl, 2.7 mM KCl, and 5 mM DTT), lysed by French press, and cleared by centrifugation at 40,000*g* for 30 min. The cleared cell lysate was incubated with glutathione sepharose resin (GE healthcare), and unbound proteins were removed by extensive washing with GST-binding buffer. Bound proteins were eluted by using a linear gradient of 0 mM to 15 mM reduced glutathione with GST-elution buffer (50 mM Tris, pH 8.0, 200 mM NaCl, 5 mM DTT, 15 mM reduced glutathione). Fractions containing GST-tagged IκBα were then dialyzed against TEV cleavage buffer (50 mM Tris, pH 8.0, 200 mM NaCl, 5 mM DTT), and the GST tag was removed from full-length IκBα by incubation with TEV protease prepared in house as described previously (37). After cleavage, the protein fractions were applied to a MonoQ anion exchange column (GE healthcare) and washed with MonoQ binding buffer (50 mM Tris, pH 7.6, 100 mM NaCl, 2 mM DTT). IκBα was then eluted by using a linear gradient of 100 mM to 1.5 M NaCl with MonoQ elution buffer (50 mM Tris, pH 7.6, 1.5 M NaCl, 2 mM DTT). Fractions containing IκBα were then applied to a Superdex 200 gel filtration column (GE healthcare) equilibrated with size exclusion buffer (100 mM Tris, pH 7.6, 200 mM NaCl, 2 mM DTT, 0.4 mM EDTA). Fractions containing full-length IκBα were pooled, concentrated, and dialyzed against storage buffer. All full-length IκBα amino acid substitution mutants were expressed and purified by using the same methods. Full-length IκBα and all IκBα amino acid substitution mutants were determined to be >95% pure on the basis of Coomassie staining of SDS-PAGE gels (38, 39).

### Expression and purification of GST-IκBα(1–54) fusion protein substrates

The GST-IκBα(1–54) fusion protein was expressed from plasmid pDBhisGST(TEV) -IkBα(1–54) in *E. coli*. This fusion protein possessed an N-terminal hexahistidine affinity tag, followed by a GST tag, a TEV protease cleavage site, and residues 1 to 54 of IκBα. The GST-IκBα(1–54) fusion protein was expressed in BL21 (DE3) pLysS at 37 °C for 3 h. The cells were then harvested, suspended in Ni binding buffer (50 mM Na_2_HPO_4_, pH 8.0, 300 mM NaCl), lysed using a French press, and cleared by centrifugation at 40,000*g* for 30 min. The cleared cell lysate was then incubated with Ni-NTA resin, and unbound proteins were removed by washing with Ni binding buffer. Bound proteins were eluted by using a linear gradient of 0 mM to 250 mM imidazole with Ni elution buffer (50 mM Na_2_HPO_4_, pH 8.0, 300 mM NaCl, 250 mM imidazole, 2 mM DTT). Fractions containing GST-IκBα(1–54) were concentrated and applied to a Superdex 200 gel filtration column equilibrated with size exclusion buffer. Fractions containing GST-IκBα(1–54) were then pooled, concentrated, and dialyzed against storage buffer as described for full-length IκBα above. The GST-IκBα(1–54) fusion protein substrate and all GST-IκBα(1–54) amino acid substitution mutants were determined to be >95% pure on the basis of Coomassie staining of SDS-PAGE gels.

### Pre–steady-state kinetic measurements of full-length IκBα phosphorylation catalyzed by constitutively active IKKβ

All kinetic assays were performed in kinase reaction buffer (50 mM Tris–Cl, pH 7.6 at 30 °C, 50 mM NaCl, 10 mM MgCl_2_, 1 mM DTT, 10% glycerol, 0.1 mM EDTA) at 30 °C. The reported concentrations are final after mixing all components. A preincubated solution containing IKKβ (S177E, S181E) (3 μM) and full-length IκBα (0.75 μM) was rapidly mixed with a solution containing [γ-^32^P]ATP (2.5 μM to 500 μM, 125 Ci/mole) and a linearized, 5′-[^32^P]-radiolabeled pBlueScript loading control (250 CPM/μl). The reaction mixtures were quenched at various times by the addition of EDTA to a final concentration of 375 mM. Reactions were carried out by using a rapid-chemical quench flow apparatus (KinTek). Reaction products were resolved by using SDS-PAGE (12% polyacrylamide) and quantified by using a Typhoon TRIO (GE Healthcare). The detected amount of [^32^P]-radiolabeled IκBα was normalized to the radiolabeled pBlueScript internal standard at each time point, and the normalized data was plotted as a function of time. The time courses of product formation at each nucleotide concentration were fit to biphasic Equation 1 (Equation 1) by using the nonlinear regression program, KaleidaGraph (Synergy Software),

(1) $$\left[product\right]=A\left([1—\exp(-k_{e}t)]+k_{l}t\right)$$

where *A* is the exponential phase amplitude, *t* is the reaction time, *k*_e_ is the exponential phase rate, and *k*_l_ is the linear rate (40, 41, 42). The *k*_*e*_ values were then plotted as a function of ATP concentration and hyperbolic Equation 2 (Equation 2) was used to acquire the maximum observed rate (*k*_p_) of IκBα phosphorylation catalyzed by IKKβ (S177E, S181E) and the binding affinity of ATP for the IKKβ•IκBα complex (*K*_d_) (43).

(2) $$k_{e}=k_{p}\left[ATP\right]⁄\left(\left[ATP\right]+k_{d}\right)$$

### Comparison of IKKβ-catalyzed phosphorylation of IκBα and IκBα amino acid substitution mutants

A preincubated solution containing IKKβ (3 μM) and either full-length IκBα wt (0.75 μM) or the indicated IκBα amino acid substitution mutant (0.75 μM) was rapidly mixed with a solution containing [γ-^32^P]ATP (500 μM, 125 Ci/mole) and a linearized, 5′-[^32^P]-radiolabeled pBlueScript loading control (250 CPM/μl). The reaction mixtures were quenched and product formation was quantified as described above. The time courses of full-length IκBα phosphorylation were then fit to biphasic Equation 1 (Equation 1).

The pre–steady-state kinetic parameters of the GST-IκBα(1–54) substrates were determined as described for the full-length IκBα substrates above. However, the IKKβ phosphorylation of the GST-IκBα(1–54) substrates lacked a clearly defined linear rate. Therefore, the time courses of GST-IκBα(1–54) product formation were fit to single-exponential Equation 3 (Equation 3), where *A* is the exponential phase amplitude, *t* is the reaction time, and *k*_e_ is the observed exponential phase rate.

(3) $$\left[product\right]=A\left[1—\exp\left(-k_{e}t\right)\right]$$

## Data availability

The article contains all data described within the text.

## Conflict of interest

The authors declare that they have no conflicts of interest with the contents of this article.
